# Improvement of Olfactory Function With High Frequency Non-invasive Auricular Electrostimulation in Healthy Humans

**DOI:** 10.3389/fnins.2018.00225

**Published:** 2018-04-24

**Authors:** Ashim Maharjan, Eunice Wang, Mei Peng, Yusuf O. Cakmak

**Affiliations:** ^1^Department of Anatomy, School of Biomedical Sciences, University of Otago, Dunedin, New Zealand; ^2^Department of Food Science, University of Otago, Dunedin, New Zealand; ^3^Brain Health Research Centre, Dunedin, New Zealand; ^4^Medical Technologies Centre of Research Excellence, Auckland, New Zealand

**Keywords:** vagal nerve stimulation, olfaction, near-infrared spectroscopy, orbitofrontal cortex, non-invasive electrostimulation

## Abstract

In past literature on animal models, invasive vagal nerve stimulation using high frequencies has shown to be effective at modulating the activity of the olfactory bulb (OB). Recent advances in invasive vagal nerve stimulation in humans, despite previous findings in animal models, used low frequency stimulation and found no effect on the olfactory functioning. The present article aimed to test potential effects of non-invasive, high and low frequency vagal nerve stimulation in humans, with supplementary exploration of the orbitofrontal cortex using near-infrared spectroscopy (NIRS). Healthy, male adult participants (*n* = 18) performed two olfactory tests [odor threshold test (OTT) and supra-threshold test (STT)] before and after receiving high-, low frequency vagal nerve stimulation and placebo (no stimulation). Participant's olfactory functioning was monitored using NIRS, and assessed with two behavioral olfactory tests. NIRS data of separate stimulation parameters were statistically analyzed using repeated-measures ANOVA across different stages. Data from olfactory tests were analyzed using paired parametric and non-parametric statistical tests. Only high frequency, non-invasive vagal nerve stimulation was able to positively modulate the performance of the healthy participants in the STT (*p* = 0.021, Wilcoxon sign-ranked test), with significant differences in NIRS (*p* = 0.014, *post-hoc* with *Bonferroni correction*) recordings of the right hemispheric, orbitofrontal cortex. The results from the current article implore further exploration of the neurocircuitry involved under vagal nerve stimulation and the effects of non-invasive, high frequency, vagal nerve stimulation toward olfactory dysfunction which showcase in Parkinson's and Alzheimer's Diseases. Despite the sufficient effect size (moderate effect, correlation coefficient (r): 0.39 for the STT) of the current study, future research should replicate the current findings with a larger cohort.

## Introduction

The olfactory system plays a fundamental role in our interactions with the environment, from detecting hazards to determining food consumption (Doty, 2012; Huart et al., 2013). Interconnected olfactory areas are responsible for various processes of the central nervous system in relation to smell, including memory (Velayudhan et al., 2013), spatial navigation (Alves et al., 2014), pleasure, mood and sensation (Sobel et al., 2003; Katata et al., 2009). Within the olfactory system, the olfactory bulb (OB) is the first communication structure responsible for processing smell. The olfactory nerve fibers, after arriving at the cranial cavity, connect to the mitral and tufted cells of the OB, and through the olfactory tract and olfactory tubercle, project to the olfactory cortex (Powell et al., 1965; Savic et al., 2000). The olfactory tract projects to the primary olfactory (cerebral) centers which includes the entorhinal cortex, piriform cortex and the parahippocampal gyrus, and then to the secondary olfactory (cerebral) centers which includes the hypothalamus, thalamus and the orbitofrontal cortex (OFC) (Savic et al., 2000; Sobel et al., 2003; Katata et al., 2009). An integral component of the olfactory system, the OFC, functions as the main neocortical projection, acting as a relay station for all arriving afferent inputs from numerous olfactory areas and sending feedback accordingly (Gottfried, 2006; Patel and Pinto, 2014).

Approximately half of the elderly population between the age of 65 and 80 years present olfactory impairments (Doty et al., 1984b; Duffy et al., 1995; Murphy et al., 2002). In addition, olfactory dysfunction is found to be a common complaint at the early stages of neurological diseases such as Alzheimer's disease (AD) and Parkinson' disease (PD), with more than 90% prevalence and higher incidence in men (Duff et al., 2002; Doty, 2012; Alves et al., 2014). Particularly with AD, odor discrimination (Fusetti et al., 2010) and identification abilities (Rezek, 1987; Motomura and Tomota, 2006), are impaired from the earliest stage of the disease. The presence of olfactory impairments at the early stage of AD has been linked to several factors occurring initially at the entorhinal cortex, one of the main terminals in the primary olfactory cortex. This includes the pathological compounds of AD (tau and amyloid precursor protein) (Naslund et al., 2000; Desikan et al., 2012), alongside reduction of cerebral blood volume (Khan et al., 2014) and thinning of cortical area (Fennema-Notestine et al., 2009; Karow et al., 2010; Velayudhan et al., 2013). Impairments in the entorhinal cortex is also seen as the link to olfactory dysfunction in the associated interconnected cortical regions (Khan et al., 2014).

In PD, impairments in odor detection (Doty, 2012), odor identification, odor discrimination (Mesholam et al., 1998) and olfactory supra-threshold measures (Doty and Kamath, 2014), are one of the earliest non-motor symptoms, preceding motor symptoms by several years (Ross et al., 2008; Doty, 2012). Impairments in PD are associated with the presence of lewy bodies, which represent distinctive inclusion bodies that underlie neuroanatomical dysfunctions in this condition. Lewy bodies are present from the earliest stage of PD, identified in the olfactory nucleus and the OB, but also in the dorsal nucleus of the vagus nerve (DMV) (Li et al., 2016). The spread of lewy bodies throughout the stages of PD, initially begins at the DMV, then spreads throughout the brainstem nuclei to the substantia nigra-pars compacta and finally the cortex (Braak et al., 2003; Doty, 2012; Li et al., 2016). This evidence points to an important connection between the vagus nerve and the olfactory processing areas of the cortex which is responsible for the early development of olfactory dysfunction in PD.

García-Díaz et al. (1984) conducted the very first study on animal models to demonstrate the capacity of invasive vagus nerve stimulation (VNS) in modulating the neuronal activity of the OB. This study showed that the ipsilateral OB neurons exhibited an increase in firing activity under high frequency (80 Hz) VNS but not under low frequency (20–40 Hz) VNS. In subsequent studies, invasive VNS in human patients with medically intractable epilepsy (Kirchner et al., 2004) and therapy-resistant depression (Sperling et al., 2011), used low frequency stimulation, where no significant effects of VNS on functional olfactory tests were found. Indeed, research has not yet addressed the question of whether high frequency VNS, as demonstrated in previous research on animal models (García-Díaz et al., 1984), is capable of modulating the olfactory performance in humans.

In a recent study, Frangos et al. (2015) has demonstrated that non-invasive VNS of the auricular branch of the vagus nerve via the left cymba conchae of the external ear, produced similar effects to invasive VNS. This finding highlights the potential benefits of using non-invasive VNS, similar to the application of invasive VNS in previous studies (García-Díaz et al., 1984; Kirchner et al., 2004; Sperling et al., 2011). To explore the potential benefits of high frequency VNS, based on a previous study using invasive VNS in animal models (García-Díaz et al., 1984), the present study aimed to examine the potential effects of non-invasive, high and low frequency VNS in humans, with supplementary exploration of OFC activation using near-infrared spectroscopy (NIRS).

## Methods

### Participants

A total of 18 Caucasian male, healthy, non-smokers participated in this study (age range = 21–38 years, mean: 24.55 years, standard deviation: 3.8 years). The participants were asked to refrain from food and non-water beverage for 2 h prior to the experiment and abstain from applying any fragrance product/s on the day of the study. This study was carried out in accordance with the recommendations of ‘Otago Human Participants Ethics Committee’ with written informed consent from all subjects. All subjects gave written informed consent in accordance with the Declaration of Helsinki. The study is approved by Otago Human Participants Ethics Committee (Reference: H16/148) and registered to the Australian New Zealand clinical trials registry (ANZCTR; registration ID: ACTRN12617000034336).

### Procedure

**Figure 1:**
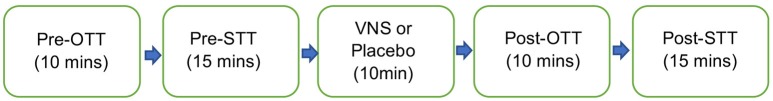
Schematic sequence of each stage of the experiment. OTT, Odor Threshold Test; STT, Supra-Threshold Test. A brief 5-min introduction, prior to the experiment comprised of obtaining participant's consent and device set up.

To measure and compare the participants in this study with the standard criteria required to meet the normative healthy responses for odor identification and memory, put forward by Doty et al. (1984a), odor identification test (Sensonics International, Haddon Heights, NJ 08035 USA; Table 1) and odor discrimination/memory test (Sensonics International, Haddon Heights, NJ 08035 USA) were completed after the placebo session. Each participant attended three, 60 min sessions with a minimum of 24 h apart. The experimental room consists of an isolated environment where no additional olfactory or visual stimuli representing food or providing any distractions were present. The experimental room kept at steady temperature (23 ± 1°C). The participants were informed not to sniff during the olfactory tests to eliminate the potential effects of sniffing. The participant was seated opposite to the experimenter performing the olfactory tests and signed consent and exclusion criteria forms to qualify the participant for the study. A brief rundown of the experimental stages was given to the participant and a second experimenter conducted the stimulation (enforcing the double-blind design). Odor threshold test (OTT) and supra-threshold test (STT) were performed before and after VNS or placebo stimulation. Figure 1 displays the experimental design of the current article. The current study followed a within participant design. In each session, participants were randomly assigned to one of the three experimental conditions: high frequency VNS, low frequency VNS or placebo session. Orders of experimental conditions were counterbalanced across the participants.

**Table 1:** Results from the pre-screening odor identification test (*n* = 18) scoring the participants in the corresponding criteria determined by their scores on the tests.

**Test score**	**Smell identification test**
6–18 (Total Anosmia)	0
19–25 (Severe Anosmia)	1
26–29 (Moderate Anosmia)	0
30–33 (Mild Microsmia)	6
34–40 (Normosmia)	11

#### Odor threshold test (OTT)

Odor Threshold Test (OTT) was performed using “Snap and Sniff Olfactory Test System” (Sensonics International, Model: 02400, Hadden Heights, NJ)—a pen-like odor dispensing device. This testing battery comprises five blank-odor pens and 15 odorant pens (phenyl ethanol; concentration ranges: 10^−2^−10^−9^, 0.5log apart). Inter-stimuli-interval was approximately 3 s; the inter-trial-interval was approximately 20 s. The experimental procedure for the OTT followed that of previous studies (Hummel et al., 1997; Ehrenstein and Ehrenstein, 1999). Individual thresholds were estimated using the standard data analysis method for the staircase test, by averaging the concentrations at which the last four points occurred (Hummel et al., 1997).

#### Supra-threshold test (STT)

A sensory discrimination test was selected to assess olfactory perception in the supra-threshold range (Lawless and Heymann, 2010). This method is referred to as the STT in the current paper. The test used Vanillin as the odorant sample (Sensient; CAS number: 121-22-5; purity: 99%). A series of aqueous solutions with varying concentrations—8, 16, and 32 ppm—were prepared using a serial dilution method while blank samples were distilled water. The STT comprised of three trials. In each trial, five samples were presented to the participants, of which two were the target and others were blank samples. Participants were asked to identify both target samples. A delay of 30 s was implemented between stimuli, and a 1 min delay between trials. One point was awarded if the participants correctly identified both target odors on each trial and a maximum of three points could be awarded.

### Vagal nerve stimulation

**Figure 2:**
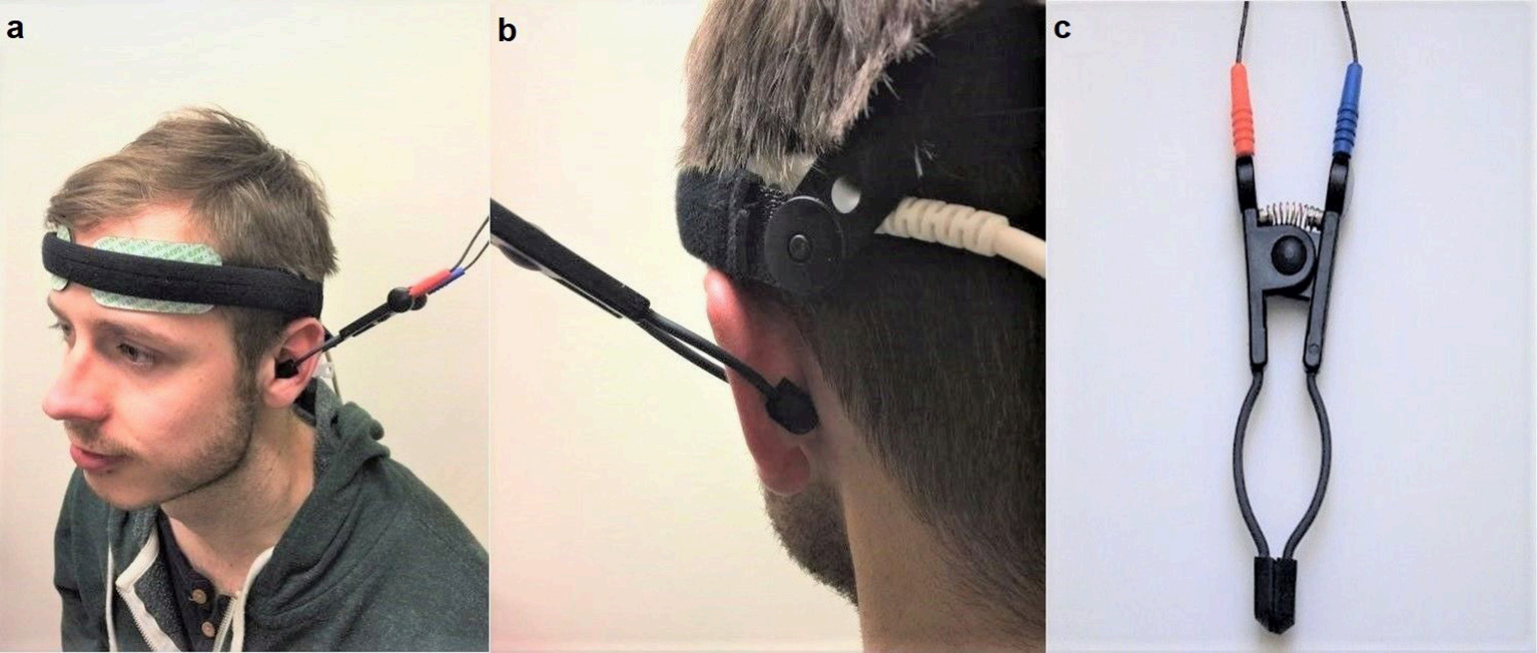
**(a)** (Left) Set up of the near-infrared spectroscopy (NIRS) electrodes on the forehead. Location and set-up of the non-invasive VNS (Auricular TENS) electrode in the cavum and cymba conchae area of the external ear. **(b)** (Right) shows location of the non-invasive VNS (auricular TENS) electrode in posterior aspect of the external ear in the current experiment. **(c)** shows the auricular TENS electrode (Schwa-Medico, France). Written informed consent was obtained from the participant for the publication of this image.

Non-invasive VNS was applied using “TENS ECO-2” (SCHWA-MEDICO, France) by the same experimenter, using transcutaneous electrical nerve stimulation (TENS) of three different parameters: high (80 Hz) frequency VNS, low (10 Hz) frequency VNS and placebo (no stimulation but the device was still attached). Previous studies on cranial nerves suggested that the parasympathetic nerve axons reach the human ear with the aid of two divisions of the auricular branch of the vagus nerve. One of the branches follows the external ear canal dispersing predominantly to the cavum and cymba conchae area of the auricular skin while the second branch travels through the posterior auricular nerve of the facial nerve, following the trajectory of the posterior auricular nerve (Schuknecht, 1974; Lang, 1983; Mulazimoglu et al., 2017). For this reason, the VNS in the current study was applied to the internal (covering the cavum concha and extending to cymba concha) and external portions of the ear (Figures 2a–c). This enforced stimulation to both branches of the auricular branch of the vagus nerve. The left vagus nerve is the preferred side of stimulation as it avoids cardiac effects, in comparison to the right vagus nerve which innervates the cardia atria (Henry, 2002). The strength of the VNS stimulation (amplitude) was between 10 and 15 milliamps and the pulse bandwidth was 180 μs in square waveform. The stimulation was only continued if there was no perceived pain by the participant.

### Near-infrared spectroscopy (NIRS)

NIRS (COVIDIEN INVOS OXIMETER, Model 5100C-PA, Mansfield, MA) was used to measure participants' activity from the OFC (set up as shown in Figure 2). Although electroencephalography (EEGs) have been used to detect different patterns of odor effects in past literature, this methodology runs the risk of misinterpretation of data due to spatial smearing, which occurs during recording of the scalp EEGs. In addition, EEGs does not provide the high spatiotemporal resolution that is supported by the NIRS (Harada et al., 2006). Several studies have displayed the effectiveness of the NIRS in monitoring the activation of the OFC under olfactory stimulus (Hongo et al., 1995; Cho et al., 1998; Edmonds et al., 1998; Ishimaru et al., 2004; Harada et al., 2006; Kobayashi et al., 2012). Specifically, Harada et al. (2006), Ishimaru et al. (2004), and Kobayashi et al. (2012), have suggested that functional magnetic resonance imaging (fMRI) localization of olfactory expression is under the lateral and anterior orbito-frontal gyri of the frontal lobe. On the basis of this information, Harada et al. (2006) placed the NIRS recording over the orbito-frontal region (secondary olfactory cortex), with the supplementary exploration of other regions (the temporal, parietal and occipital lobes) and found that oxygenated hemoglobin (HbO2) concentration increased only over the orbito-frontal region during olfactory stimulation.

Regional hemoglobin oxygen saturation (rSO_2_) monitoring NIRS systems permit the continuous and non-invasive measurement of the cerebral regional oxygen balance in the cortex. NIRS, briefly, is a useful non-invasive method of monitoring regional tissue oxygenation. By using the modified Beer-Lambert law for the light attenuation changes through the illuminated tissue, NIRS sequentially detects concentration changes of oxygenated and deoxygenated hemoglobin in the brain. As oxygenated hemoglobin concentration represents an increase in blood flow, this, in turn reflects neuronal activation measured by the NIRS (Harada et al., 2006). The INVOS monitors the cortical activity while excluding information from the skin and scalp blood flow. The INVOS system sensors uses two near-infrared light sources at 2 different wavelengths (730 and 810 nm) and two photodiode detectors at a distance. Light travels from the sensor's light emitting diode to either a proximal or distal detector in parabolic path, permitting separate data processing of shallow and deep optical signals with an algorithm of subtraction of the short travel distance of the light from the longer distance of travel in order to eliminate the contribution of the skin and scalp. The INVOS system's ability to localize the area of measurement with the process of suppressing the influences of extra-cerebrally reflected photons, called spatial resolution, has been empirically validated in human subjects (Hongo et al., 1995). It uses a clinically validated algorithm that allows absolute, real time data accuracy from the cortex with its multi-sensors on the same sensor (Hongo et al., 1995; Cho et al., 1998; Edmonds et al., 1998, 2004; Roberts et al., 1998; Higami et al., 1999; Singer et al., 1999; Yao et al., 2001; Alexander et al., 2002; Iglesias et al., 2003). INVOS measures both the venous and arterial blood in a 3:1 ratio to monitor cortical activity. In this context, INVOS measures the venous-weighted percent of rSO_2_ (VWrSO_2_) in the cortex, which provides real-time information concerning the balance of oxygen supply and demand and in turn, calculates the venous oxygen reserve (VOR) that reflects the remaining oxygen after extraction by tissues and vital organs (Hongo et al., 1995; Cho et al., 1998; Edmonds et al., 1998, 2004; Roberts et al., 1998; Higami et al., 1999; Singer et al., 1999; Yao et al., 2001; Alexander et al., 2002; Iglesias et al., 2003). This system allows a sensitive and real-time measurement of VOR (measured as VWrSO_2_). In addition, the quality of INVOS system recording can be assessed by inspection of the signal strength index (SSI) for each channel by 5 unit bar scale system. Any stable SSI signal display of >1 bar is reported to be strong and stable enough to generate an accurate VWrSO_2_ [Chapter 6.4.16 and 11.7.4 in the INVOS NIRS 5100c manual (Covidien, 2013)]. The SSI bar were monitored throughout the recordings to ensure the highest quality of signal (5/5 SSI). We used disposable INVOS electrodes for each participant to ensure hygiene and data quality. In addition to self-adhesive feature of the disposable INVOS sensors, we also improved the stabilization of the electrodes and cables with a rigid head band (Figure 2).

In the context of the extensive literature and clinical validation studies, we used FDA approved INVOS NIRS for the present study. With the NIRS, each section of the experiment was mapped, to ensure that each part of the experiment (*experimental stages shown in* Figure 1) could have an average (mean) recording of VWrSO_2_ (that represents VOR) for the participant from the left and right hemisphere of the OFC.

### Data analysis

Data from the NIRS device was transferred to the INVOS software which accommodated data presentation. We used average (mean) recordings of VWrSO_2_ data of specific time periods marked for each segment of each session which was in line with the previous research (Cho et al., 1998; Murkin et al., 2007). Repeated-measures analyses of variance (ANOVA) were applied separately to data obtained from three stimulation parameters (High VNS, low VNS and placebo) for assessing VWrSO_2_ changes in the left and right hemispheric OFC across stages of the experiment (pre-stimulation, stimulation and post-stimulation) within participants (*n* = 18). Specifically, the repeated-measures ANOVA was applied to STT and OTT periods separately in order to provide synchronized analyses of NIRS data with the functional olfactory tests and stimulation (i.e., preSTT-Stimulation-postSTT periods of NIRS and preOTT-Stimulation-postOTT periods of NIRS). In addition to this 3-stage analysis of NIRS, an additional 5-stage analysis of NIRS (preOTT-preSTT-Stimulation-postOTT-postSTT, in the order of experiment, see Figure 1) were also performed to reveal any potential influence of OTT over STT (if any). Post hoc test using pairwise comparison with *Bonferroni correction*, was applied to understand any significance at alpha level of 5% in all ANOVA tests. To insure the baseline activity was consistent across three different stimulation parameters for OTT, STT, and NIRS data, repeated measures ANOVA was also applied to data that obtained at pre-stimulation stage (Table 2).

**Table 2:** Results of the repeated-measures ANOVA that assessed the potential difference (if any) of olfactory test (OTT, STT) and also the NIRS (VWrSO_2_) data in all of the three different stimulation parameters (high- and low frequency VNS and placebo) at the pre-stimulation stage of testing.

**Olfactory tests and NIRS recordings (h = hemisphere)**	**Stimulation (H = High, L = Low and P = Placebo)**	**Mean**	**S.D**.	***F*-value DF (1, 17)**	***P*-value**
Pre-S OTT scores	H	6.02	1.41	0.099	0.889
	L	5.83	1.71		
	P	5.92	1.53		
Pre-S STT scores	H	1.55	1.20	2.006	0.152
	L	1.78	0.94		
	P	2.11	0.76		
Left-h VWrSO_2_ at Pre-S OTT	H	76.72	9.73	1.156	0.319
	L	76.23	8.48		
	P	74.72	9.84		
Right-h VWrSO_2_ at Pre-S OTT	H	76.91	9.05	2.129	0.142
	L	78.04	9.81		
	P	75.55	9.74		
Left-h VWrSO_2_ at Pre-S STT	H	76.65	9.49	1.663	0.210
	L	75.58	8.85		
	P	74.26	9.91		
Right-h VWrSO_2_ at Pre-S STT	H	77.35	10.06	2.016	0.156
	L	76.21	9.09		
	P	74.75	10.59		

*S.D., standard deviation; DF, Degrees of Freedom. STT scores range = 0–3, OTT scores range = 2–9. H, hemisphere; H, High Frequency Stimulation; L, Low frequency Stimulation; P, Placebo*.

For data obtained from the olfactory tests, normality tests using the *Shapiro-Wilk correction* (Ghasemi and Zahediasl, 2012) were performed on the differences between the scores before and after stimulation for all three parameters (High VNS, low VNS, and placebo). This test was separately applied to the STT and OTT data. Data for participants under low frequency VNS and placebo stimulation for OTT and for participants under placebo stimulation for STT, passed the normality test (*p* > 0.05). Thus, a paired sample *t*-test was performed on these data. The rest of the data (high frequency VNS for both OTT and STT tests, and low frequency VNS for STT test) failed the normality test (*p* < 0.05), so a non-parametric (Wilcoxon signed rank test- Gibbons and Chakraborti, 2011) was performed for this set of data. All the analyses were performed using SPSS software (IBM SPSS Statistics, Ver. 20, St Leonards, NSW).

## Results

### Pre-screening olfactory test results

The results from the odor identification and memory tests used to ensure that the healthy participants in the current study represented the standard criteria required to meet the normative healthy responses for odor identification (Table 1) and memory tests (Doty et al., 1984a, 1995). Seven of the eighteen participants displayed values outside of Normosmia, but none of the participants displayed total anosmia which was the exclusion criteria for the current study in line with the past olfactory research (Doty et al., 1984a, 1995). In this context, all the participants were eligible for the current study.

### OTT results with 3-stage (PreOTT-stimulation-postOTT) analysis of NIRS data

**Figure 3:**
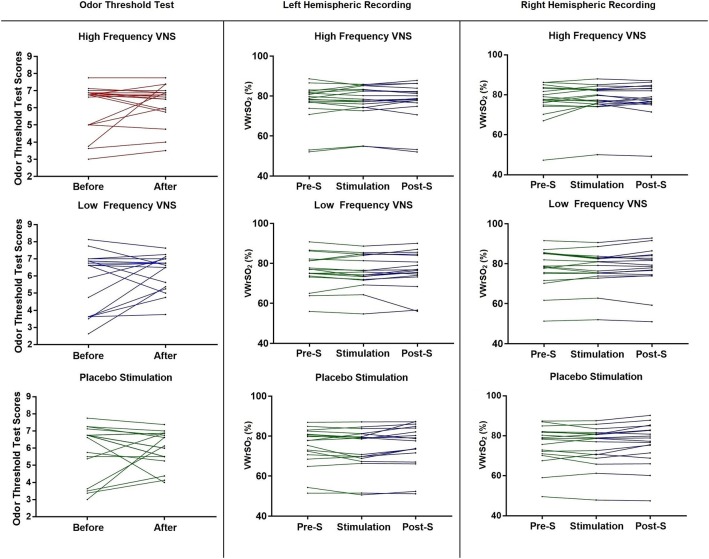
Each participant's scores, before and after each of the stimulation parameters (high frequency VNS, low frequency VNS and placebo) for the OTT, and each participant's recordings for all three stages of the experiment (pre-stimulation OTT; Pre-S, stimulation and post-stimulation OTT; Post-S) for each stimulation parameter (high frequency VNS, low frequency VNS and placebo) from both the left and the right hemispheres of the OFC, measuring venous oxygen reserve-(VWrSO_2_ %) using NIRS. OTT scores range = 2–9.

Figure 3 shows each participant (*n* = 18) results from the OTT, before and after VNS stimulation, for all three stimulation parameters (high- and low frequency VNS and placebo). There was no significant difference in the performance of the OTT after VNS stimulation under any of the three stimulation parameters [high frequency VNS, *p* = 0.523 (Wilcoxon signed-rank test); low frequency VNS, *p* = 0.186 (paired sample *t*-test); placebo stimulation, *p* = 0.904 (paired sample *t*-test)]. Individual results are provided in Figure 3. In the NIRS recording of the OFC in the left and right hemispheres during the OTT, there were no significant differences in VWrSO_2_ (%) under all three stimulation parameters in all three stages of the experiment (Pre-S, S, Post-S) (left hemisphere High frequency VNS, *p* = 0.643; left hemisphere Low frequency VNS, *p* = 0.570; left hemisphere Placebo, *p* = 0.061; right hemisphere High frequency VNS, *p* = 0.233; right hemisphere Low frequency VNS, *p* = 0.565; right hemisphere Placebo, *p* = 0.098). Individual results are presented in Figure 3.

### STT results with 3-stage (PreSTT-stimulation-postSTT) analysis of NIRS data

**Figure 4:**
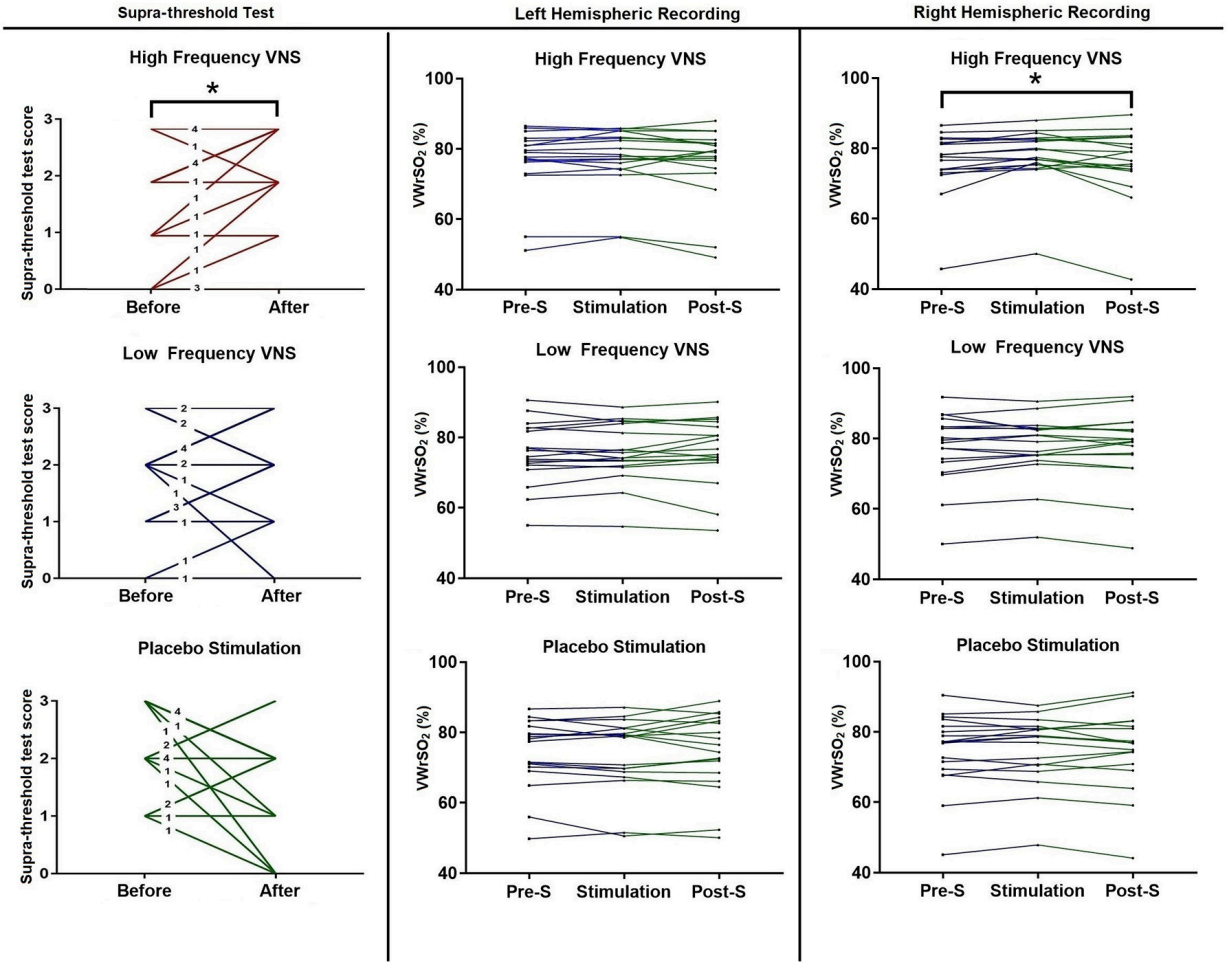
Each participant's scores, before and after each of the stimulation parameters (high frequency VNS, low frequency VNS and placebo) for the STT, and each participant's recordings for all three stages of the experiment (pre-stimulation STT; Pre-S, stimulation and post-stimulation STT; Post-S) for each stimulation parameter (high frequency VNS, low frequency VNS and placebo) from both the left and the right hemispheres of the OFC, measuring venous oxygen reserve (VWrSO_2_ %) using NIRS. The numbers in each line for the STT scores represents the number of cases that represent the corresponding result. STT scores range = 0–3. ^*^Statistically significant (*p* < 0.05).

Figure 4 shows each participant (*n* = 18) results from the STT, before and after VNS stimulation, for all three stimulation parameters (high- and low frequency VNS and placebo). There were significant differences in the STT scores after high frequency VNS [*p* = 0.021 (Wilcoxon signed-rank test), correlation coefficient (r): 0.39] but not under the low frequency VNS [*p* = 0.439 (Wilcoxon signed-ranked test) or placebo stimulation (*p* = 0.083 (paired sample *t*-test)]. Individual results are presented in Figure 4. In the NIRS recording of the OFC in the right hemisphere, there were significant differences in VWrSO_2_ (%) between the three stages of the experiment after high frequency VNS (*p* = 0.031). *Post-hoc* tests using the *Bonferroni correction* revealed that there were significant differences in NIRS OFC recording on the right hemisphere between Pre-S stage and S stage (*p* = 0.014). There were no significant differences in VWrSO_2_ (%) between the three stages of the experiment after high frequency VNS in the left hemisphere (*p* = 0.253), or after low frequency VNS in both hemispheres (left hemisphere, *p* = 0.693; right hemisphere, *p* = 0.732) or after placebo (left hemisphere, *p* = 0.697; right hemisphere, *p* = 0.849). Individual results are presented in Figure 4. Additional scatterplot chart of each individual participant, before and after stimulation parameters (high frequency VNS, low frequency VNS and placebo stimulation), STT scores and the corresponding right hemispheric OFC NIRS recordings (pre-stimulation, stimulation and post-stimulation) are provided as Supplementary Files 1–3.

### 5-stage (preOTT-preSTT-stimulation-postOTT-postSTT) analysis of NIRS

An additional ANOVA analysis of the 5-stage NIRS periods (preOTT-preSTT-Stimulation-postOTT-postSTT, Figure 1.) in the order of experiments for all stimulation parameters (low, high and placebo) for the right and left hemisphere demonstrated a significance only in the high frequency stimulation group for the right hemisphere (p:0.037) and pairwise comparisons demonstrated the significance (p:0.046, *post-hoc* tests using the *Bonferroni correction*) only in between the preSTT and stimulation periods of NIRS in this group, similar to the results derived from the 3-stage (preOTT-Stimulation-PostOTT and preSTT-Stimulation-postSTT) analysis of NIRS.

### Pre-stimulation, intergroup differences from OTT and STT, and respective NIRS recordings from OFC

Table 2 displays results of the repeated-measures ANOVA that assessed the potential difference (if any) of olfactory test (OTT, STT) and also the NIRS (VWrSO_2_) data in all of the three different stimulation parameters (high, low frequency VNS and placebo) at the pre-stimulation stage of testing. There was no significant difference in the pre-stimulation stage for both olfactory tests and NIRS VWrSO_2_ (%) data under any stimulation parameter (Table 2).

## Discussion

The current study tested the effects of non-invasive, high and low frequency VNS on olfactory sensory tests in healthy adult participants, with the addition of functional imaging technique in the OFC. To our knowledge, this was the first data suggesting non-invasive high frequency (80 Hz) VNS can positively modulate olfactory performance in healthy participants. Indeed, olfactory performance was improved after the implementation of high frequency VNS in the STT, although not in the OTT. In contrast, non-invasive low frequency (10 Hz) VNS and placebo did not show any effect on modulating performance in the olfactory sensory tests. These results were supported by increased VWrSO_2_ in the NIRS recordings of the OFC in the right hemisphere.

In the current article, non-invasive, low frequency VNS did not change the ability to influence olfactory function in the healthy participants. This result is in line with previous research using invasive, low frequency VNS on humans (Kirchner et al., 2004; Sperling et al., 2011). It should be stated that previous studies in humans were performed on patients with medically intractable epilepsy (Kirchner et al., 2004) or patients with therapy-resistant depression (Sperling et al., 2011), not healthy controls. The use of invasive low frequency VNS in these previous articles fitted the stimulation parameters for patients with medically intractable epilepsy (Kirchner et al., 2004) and therapy-resistant depression (Sperling et al., 2011) instead of stimulation parameters that could potentially impact olfactory function. As the previous articles in VNS used invasive procedures on patients requiring VNS therapy rather than healthy controls, it was infeasible to access different VNS of higher frequencies. This includes the exploration of high frequency VNS of 80 Hz, which in previous animal models showed to be effective in modulating the activity of the OB (García-Díaz et al., 1984).

In contrast to the low frequency VNS, non-invasive, high frequency VNS was demonstrated to result in significant improvements on STT performance. This is in support of previous research where low and high frequency stimulation had opposite autonomic nerve system responses in previous human studies of peripheral nerve stimulation (Cakmak et al., 2008, 2016, 2017; Zhao, 2008). Previous studies have reported that different olfactory measures such as the odor identification test, odor recognition test or the OTT could be processed under the same cortical/subcortical areas associated with olfaction (Mesholam et al., 1998). Furthermore, in healthy participants, olfactory functioning from the OTT was highly correlated with that of the odor identification test (Doty et al., 1989, 1994). However, in both these articles, there were no measures observing the olfactory functioning through the STT.

In a recent study (Hedner et al., 2010), cognitive variables from different types of olfactory tests such as the OTT, odor discrimination test and the odor identification test were observed. They suggested that OTT is governed by sections of the cortex that is responsible for low-level perceptual function (such as the volume of the OBs related to the peripheral sensory input of olfactory processes) whereas tests of odor discrimination/recognition/identification tests poses more cognitive demands and are represented in cortical areas for higher-level perceptual functions. This could explain the variability seen in the current study where no expression of change was seen in the OTT scores after all stimulation parameters, nor was there any chance in the OFC recording while STT scores improved after high frequency stimulation, alongside the increase of OFC activity on the right hemisphere (represented through the increased VWrSO_2_). Future research is required to observe olfactory structures that are engaged during the STT that differentiates it from separate olfactory functional tests mentioned previously. This could represent the results from this current article, where only the STT displayed improvements after high frequency VNS. The investigation of STT during VNS or straight after VNS could also be performed in a future study to observe if there would be a more prominent effect on the STT scores than what was observed after the stimulation in the current article. However, the results from the current study showed a statistical significance in the NIRS recordings between pre-stimulation and stimulation stages in the pairwise comparison using *Bonferroni correction* for the STT, but this was not the case for the stimulation and the post-stimulation stages. Therefore, this may exclude the potential washing out effect of auricular vagal nerve stimulation on STT scores. In addition, 5-stage analysis of NIRS data demonstrated a significance only in high frequency stimulation group on the right hemisphere with an indication of the difference between the preSTT and stimulation periods of NIRS. However, there was no significant difference between the OTT and STT periods of NIRS data, suggesting that the OTT test did not exert influence on STT in terms of NIRS recording.

**Figure 5:**
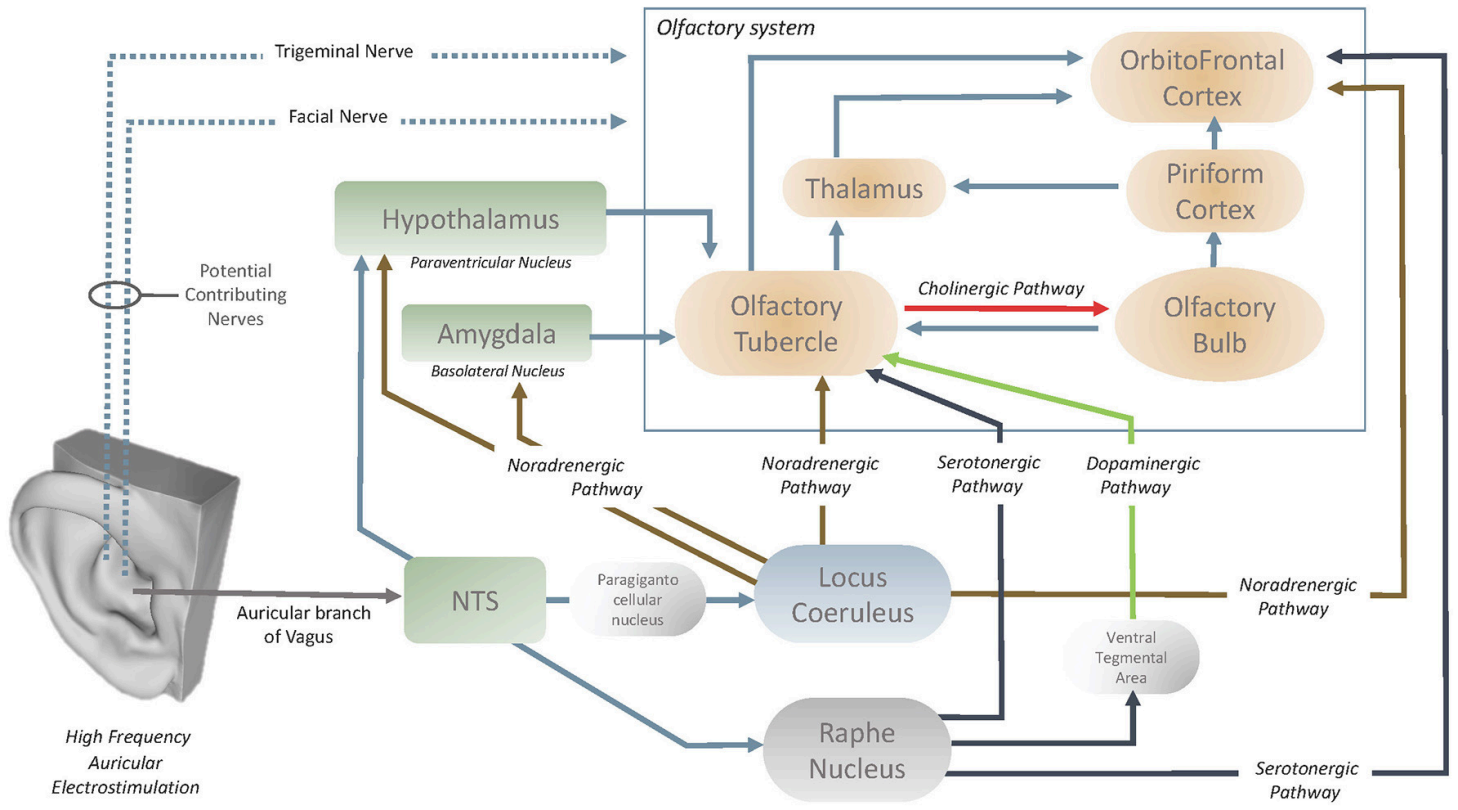
Pathways from the auricular vagus nerve to the olfactory system.

Exploration of the neuroanatomical structures that are affected by high frequency VNS would aid in the understanding of how the olfactory centers are influenced by the vagus nerve. In the past literature, García-Díaz et al. (1984) reported that only high frequency VNS increased the activity of the OBs, with the potential effect of VNS through the periglomerular layer of OBs. It is also understood that the cholinergic elements of the OBs originate in the olfactory tubercle (OT) (Shute and Lewis, 1967) and it has been demonstrated (Gervais, 1979) that the OT modulates the centrifugal control over the OB (Figure 5). This therefore also implies the presence of an oligosynaptic pathway by which the vagus nerve influences the OB via the OT. OT is heavily innervated by numerous neuromodulatory centers in the brain and brainstem (Wesson and Wilson, 2011). The OT receives noradrenergic fibers of locus Coeruleus (LC) and serotonergic fibers of Raphe nucleus (Solano-Flores et al., 1980; Guevara-Guzman et al., 1991). The last but not the least, Raphe nucleus also acts on OT through the dopaminergic pathway that passes through the ventral tegmental area (Pierce et al., 1976; Hervé et al., 1987). Both the LC (via paragigantocellular nucleus) (Chandler et al., 2014) and raphe nucleus have afferents from nucleus of the tractus solitarius (NTS) and it has been demonstrated that both structures can be stimulated with auricular vagal nerve stimulation (Sawchenko, 1983; Ruggiero et al., 2000; Mello-Carpes and Izquierdo, 2013; Frangos et al., 2015). NTS has a role as the hub to convey the neuromodulatory effects of the auricular vagal nerve stimulation (Frangos et al., 2015). In the context of underlined numerous interconnections of the OT, it can be considered that OT can easily be modulated with auricular vagal nerve stimulation and it is considered as likely the contributor to state-dependent olfactory processing (Wesson and Wilson, 2011; Figure 5).

OFC is the secondary order station of olfactory system that can be modulated by OT or directly by LC or raphe nucleus (Kannan and Yamashita, 1985; Mooney et al., 1987; Ikemoto, 2007; Price, 2010; Wesson and Wilson, 2011; Chandler et al., 2014; Zhou et al., 2015). LC and Raphe nucleus acts directly on the OFC via noradrenergic and serotonergic pathways respectively (Wesson and Wilson, 2011; Chandler et al., 2014; Zhou et al., 2015). In addition to the OT, OFC is also prone to modulation by auricular vagal nerve stimulation. The vagus nerve could also stimulate the NTS-locus Coeruleus (or raphe nucleus)-OFC pathways (Kannan and Yamashita, 1985; Samuels and Szabadi, 2008; Wesson and Wilson, 2011; Mello-Carpes and Izquierdo, 2013; Chandler et al., 2014; Frangos et al., 2015). Furthermore, LC can also act on basolateral nucleus of the amygdala and/or paraventricular nucleus of the hypothalamus (Samuels and Szabadi, 2008; Mello-Carpes and Izquierdo, 2013). Both the basolateral nucleus of the amygdala and paraventricular nucleus of the hypothalamus acts on the OT (Samuels and Szabadi, 2008; Wesson and Wilson, 2011). The NTS can also project directly to the hypothalamic paraventricular nuclei which act on the OT (Kannan and Yamashita, 1985; Wesson and Wilson, 2011; Zhou et al., 2015). In summary, OT, OB and OFC can be modulated via serotonergic, noradrenergic, cholinergic and dopaminergic pathways by auricular vagal nerve stimulation (Figure 5).

In past research on rat-animal models (Ziomber et al., 2012), the use of invasive, low frequency VNS had reported inhibitory effects on the dopamine system in the nucleus accumbens, frontal cortex, ventral tegmental area and the striatum. Ziomber et al. (2012) indicated that invasive low frequency VNS displayed similar effects on dopaminergic impairments to vagotomy. Previous studies have also linked the dopaminergic system with olfactory function, indicating that the dopaminergic modulation can alter odor detection thresholds and odor discrimination abilities (Ziomber et al., 2012). However, the use of low frequency, non-invasive VNS in the current article did not have any effects on olfactory performance. It was hypothesized that although low-frequency VNS, through the potential modulation of the dopaminergic system, leads to an impaired VN function, in contrast, high-frequency stimulation could potentially have the opposite effects (Cakmak et al., 2008, 2016, 2017; Zhao, 2008). This hypothesis is supported by the current findings, where only high frequency VNS was capable of improving olfactory performance in the STT. These results in the current article implore future research to encapsulate the exact mechanism of action of how different frequencies of non-invasive VNS affects the dopaminergic system. This could explain the results from both stimulation parameters in the current article, and detail how each stimulation parameter affects the dopaminergic system.

The use of functional imaging techniques, alongside VNS is a crucial step toward understanding the effects of VNS on the olfactory system. In the current article, NIRS was used as the functional imaging technique to observe VNS effects on the OFC. The OFC is considered the communication station of olfaction in the brain (Patel and Pinto, 2014) with a direct pathway to the neocortex. The OFC is involved in multiple complex sensory pathways that provide input from most of the major sensory and limbic structures, highlighting its role in multisensory integration (Shepherd, 2009). Previous reports using functional brain imaging techniques (Handforth et al., 1998; Henry et al., 1999; Bohning et al., 2001; Lomarev et al., 2002) have also reported that invasive VNS stimulation of 20–30 HZ caused significant increases in blood flow in the bilateral OFC, which did not occur in the invasive VNS stimulation of 1 Hz. In contrast to the previous studies using VNS of 20–30 Hz, (Handforth et al., 1998; Henry et al., 1999; Bohning et al., 2001; Lomarev et al., 2002; Kirchner et al., 2004; Sperling et al., 2011; Frangos et al., 2015) but in support of VNS using 1 Hz (Bohning et al., 2001; Lomarev et al., 2002), the current article did not present any significant effects under VNS using 10 Hz in the OFC. In comparison, high frequency VNS of 80 Hz, displayed significant differences in the contralateral-right hemispheric OFC. In summary, the spectrum of different frequencies of VNS results in differentiating effects on the OFC, with 1–10 Hz presenting no effect on either hemisphere of the brain, 20–30 Hz presenting bilateral OFC activation and 80 Hz presenting contralateral OFC activation. This could be the key to understanding why only high frequency VNS of 80Hz was effective in modulating olfactory performance in the current article.

In the meta-analysis and review (Li et al., 2016), it was reported that PD patients presented a significantly larger OB volume on the right hemisphere, indicating the presence of lateralized differences between the two hemispheres. In support, several articles have indicated that the right hemisphere is more important for higher-order processing of smell sensation that the left hemisphere (Zatorre et al., 1992; Jones-Gotman and Zatorre, 1993; Hummel et al., 1995). Functional imaging studies attribute the right-side lateralized differences to odors inducing increased activation within the right piriform cortex and OFC, which are core anatomical structures in olfactory processing (Zatorre et al., 1992). The current article is in support of the aforementioned studies, as the improved olfactory performance was attributed to increased activation of the contralateral, right hemispheric OFC. With the support from the results of the current article, it could be acknowledged that improvements in the STT under high frequency VNS was through the activation of the contralateral OFC activation, in contrast to previous reports of bilateral OFC activation through low frequency VNS (Handforth et al., 1998; Henry et al., 1999; Bohning et al., 2001; Lomarev et al., 2002; Kirchner et al., 2004; Sperling et al., 2011; Frangos et al., 2015).

The loss of olfactory function is very common in neurological diseases including AD, PD, vascular dementia and fronto-temporal dementia (Kovács, 2004; Alves et al., 2014; Godoy et al., 2015). The loss of olfactory functioning is an early symptom in AD and PD and is seen, not only as a pre-mediator of the disease, but a factor that could affect separate symptomology of the disease (Alves et al., 2014). It has been indicated that the higher the olfactory dysfunction is at baseline in patients diagnosed with PD, the higher the risk of developing visual hallucinations and greater cognitive dysfunction (Doty, 2012). Furthermore, loss of olfactory function in PD separates this neurological disorder from other movement disorders (Doty, 2012). A recent article reported that patients with PD had reduced STT scores in comparison with healthy controls (Doty and Kamath, 2014). It is also worth to note that the recovery of the olfactory function induces the neuroplasticity in patients with smell loss (Kollndorfer et al., 2014). In a previous study, our group (Cakmak et al., 2017) demonstrated clinically significant improvements in PD motor symptoms with high frequency (130 Hz) auricular electrostimulation including auricular vagal nerve territory. In this context, as high frequency VNS in the current article improved healthy participant's olfactory abilities in the STT, a potential beneficial effect of high frequency vagal nerve stimulation on olfactory function in early AD and PD needs to be investigated. Further exploration toward the effects of VNS on olfactory processes should use the non-invasive approach as it has not only exhibited similar effectiveness as the invasive approaches (Frangos et al., 2015) but it also, eliminates the risk associated with surgical implementation (Handforth et al., 1998; Fahy, 2010).

There are some limitations to consider in the present study. We acknowledged that there are various olfactory behavioral tasks available. While the STT was employed in the present research, future should replicate these findings with other types of tasks. In addition, NIRS monitoring was only performed to observe the OFC, therefore unable to encapsulate the entire cortex including the piriform cortex. fMRI would be convenient option to visualize entire networks of the olfactory system that could be influenced with auricular vagal nerve stimulation. A previous fMRI study with low frequency, auricular vagal nerve stimulation demonstrated alterations in numerous different brain regions (Frangos et al., 2015). In this context, a potential effect of high frequency, auricular vagal nerve stimulation on different neuronal networks cannot be excluded. However, low frequency cervical and auricular VNS has been used as a treatment option for intractable epilepsy, chronic treatment-resistant depression (Howland, 2014), pain (Ellrich, 2011; Howland, 2014), primary headaches and medication-overuse headaches (Gaul et al., 2016), and lately high frequency auricular VNS has been used by our group in Parkinson's Disease (Cakmak et al., 2017). To our knowledge, there have been no side effects reported of non-invasive VNS in the context of cortical activity and therefore, a negative effect through the use of non-invasive VNS would be a rare possibility in the context of previous research. In addition, it is also worth to mention that trigeminal and facial nerves have the potential to modulate NTS and as a consequence, the olfactory function (Escanilla et al., 2015). The anatomical connections in between the facial and trigeminal nerves were demonstrated (Cobo et al., 2017) and the auricular stimulation zone in the present study comprises both the external and the posterior aspect of the auricle as shown in Figures 2a,b. Both of these zones include potential contributions from the facial and trigeminal nerve branches, therefore potential contributions of the facial and trigeminal nerve on the observed olfactory modulatory effects of auricular stimulation cannot be excluded.

Although the NIRS monitoring have the advantages of real-time, non-invasive and continuous monitoring of changes in regional oxygen saturation, it has some limitations. Within the wavelength region of interest (730–810 nm), melanin and water are also in the light spectrum of photon absorption, fortunately it has been demonstrated that INVOS NIRS recordings were relatively unaffected by normal skin pigmentation in the adults (Misra et al., 1998; Damian and Schlosser, 2007). It is worth to note that the within-subject design of the present study also helped to ameliorate potential biasing effects (if any) due to inter-individual differences in skin color. Hair or hair follicles can produce excessive photon scattering in NIRS and it may result with artifactual low rSO_2_ (Orihuela-Espina et al., 2010). Therefore, only the clinically validated placement for the sensors (the frontal head-hair free area) were used in the present study. Skull and skin perfusion data can also alter the NIRS recordings however the NIRS-INVOS (5100c model in the present study) uses two specific wavelengths of near-infrared light to determine oxygen hemoglobin saturation in the tissue beneath the sensor, with two detectors (shallow and deep) that allows the suppression of superficial tissue. This allows accurate measurement of site-specific tissue oxygenation (Thavasothy et al., 2002; Edmonds et al., 2004; Hessel et al., 2014). Cranial bone anomalies, frontal sinus inflammation, dyshemoglobinemias like local or systemic pathologies are also reported to be the potential artifact factors for NIRS recordings (Gopinath et al., 1995; de Letter et al., 1998; Madsen et al., 2000; Sehic and Thomas, 2000; Boulos et al., 2007; McRobb et al., 2011). In addition, NIRS is also susceptible to ambient light and motion artifacts of relative movement between an optical fiber and the scalp. All of these underlined potential artifacts are reported to be resolved by fluctuating/unstable bar signal alert of SSI data quality monitoring system of the INVOS (NIRS 5100c) (Chapter 6.4.16 and 11.7.4 in the INVOS NIRS 5100c manual; Covidien, 2013). In the context of the knowledge of any stable SSI bar display of >1 is an accurate signal of WVrSO_2_ and our stable signal at 5/5 SSI bar in the present study, we can conclude that our recordings with NIRS are in highest quality of clinically validated INVOS system. It is also worth to note that we only enrolled healthy participants in our study to eliminate such potential pathological artifacts but also to interact with a healthy olfactory system. In addition, we also kept the room temperature and the position of the participants constant during the experiments for a potential effect on NIRS measurements.

Results of the present study were obtained in 18 participants in a within-subject study with three conditions—a placebo and 2 different stimulation conditions (low and high VNS). In addition to presented *p*-values for the statistical significance, the effect size [correlation coefficient (r): 0.39 for the STT test with the significant output after high frequency VNS] indicated a medium/moderate effect in the context of Cohen's thresholds for interpreting the effect size (r:0.1 small effect, r:0.3 moderate effect, r:0.5 large effect, Cohen, 1988). Future studies are needed to replicate our study with a larger cohort. In addition, the present study included only healthy male participants due to difficulties of enrolling female participants in the same period of their menstrual cycle. Future studies are also needed to investigate the potential different responses of neuromodulation on female cohorts in the context of hormonal influences over the olfactory system.

In conclusion, the present article exhibited, for the first time in human research, that non-invasive high frequency auricular vagal nerve stimulation is effective in improving the healthy adult olfactory function in the STT, accompanied by increased activation of the right hemispheric orbitofrontal cortex. The present study underlies the significance of the frequency in auricular vagal nerve stimulation to modulate olfactory function. Further studies are needed to investigate potential effects of different frequencies of vagal nerve stimulation on olfactory function and related neural networks.

## Author contributions

YC: The research concept idea; YC and MP: Study design and methodology; YC, EW and AM: Performing the experiments; AM and MP: Statistical Analysis; AM, MP and YC: Analyzing the results; AM, MP and YC: Writing the manuscript.

### Conflict of interest statement

The authors declare that the research was conducted in the absence of any commercial or financial relationships that could be construed as a potential conflict of interest.

## Supplementary material

Supplementary Files 1–3 Scatterplot graph which displays each participant's scores, before and after each stimulation parameter (high frequency VNS, low frequency VNS and placebo) for the STT scores (scores range = 0–3) in combination with participant's recordings for all three stages of the experiment (pre-stimulation STT; Pre-S, stimulation and post-stimulation STT; Post-S) for the right hemisphere of the OFC, measuring venous oxygen reserve (VWrSO_2_ %) using NIRS. In the STT score figures (Figure on top), “Red” color represents participants who improved, while on the STT right hemisphere NIRS recordings (Figure on bottom), “Red” color represents improvements (increased % in VWrSO_2_) in the stimulation stage from the pre-stimulation stage and “Orange” color represents improvements (increased % in VWrSO_2_) in the post-stimulation stage from the pre-stimulation stage. Supplementary file 1 shows the results from high frequency VNS, Supplementary file 2 shows the results from the low frequency VNS and Supplementary file 3 shows the results from the placebo stimulation.

## Abbreviations

VOR: venous oxygen reserve
OTT: odor threshold test
STT: supra-threshold test
DMV: dorsal nucleus of the vagus nerve
OFC: orbitofrontal cortex
Pre-S: Pre-stimulation
S: stimulation
Post-S: post-stimulation
VWrSO_2_: Venous Weighted percent of regional oxygen saturation.
