# Supplementary figures and images for: Improvement of Olfactory Function With High Frequency Non-invasive Auricular Electrostimulation in Healthy Humans
Source: Front Neurosci. 2018 Apr 24;12:225. doi: 10.3389/fnins.2018.00225 (PMC5928377; doi:10.3389/fnins.2018.00225)

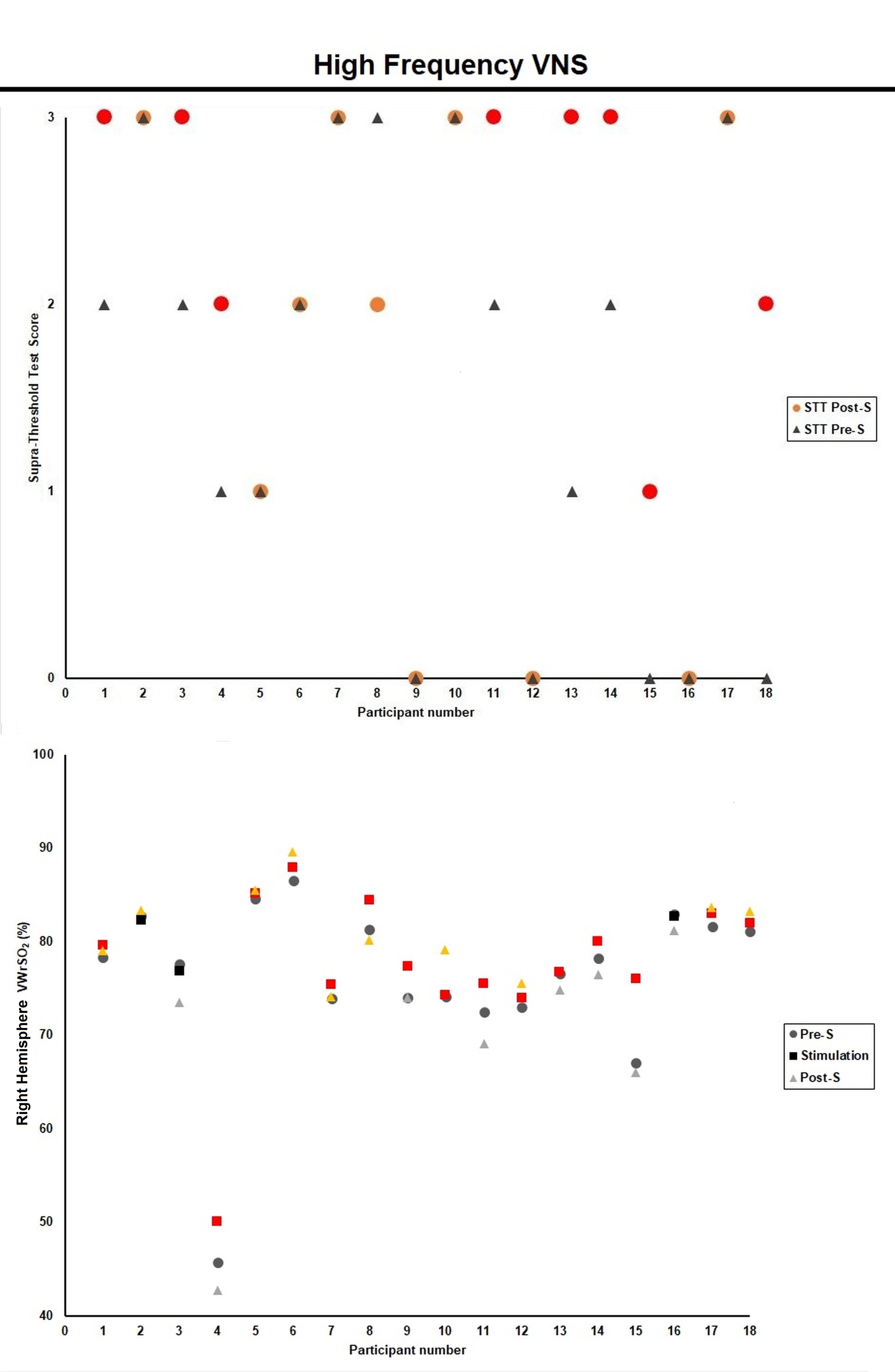

Supplement: Supplementary file 1 [file Image_1.jpeg]

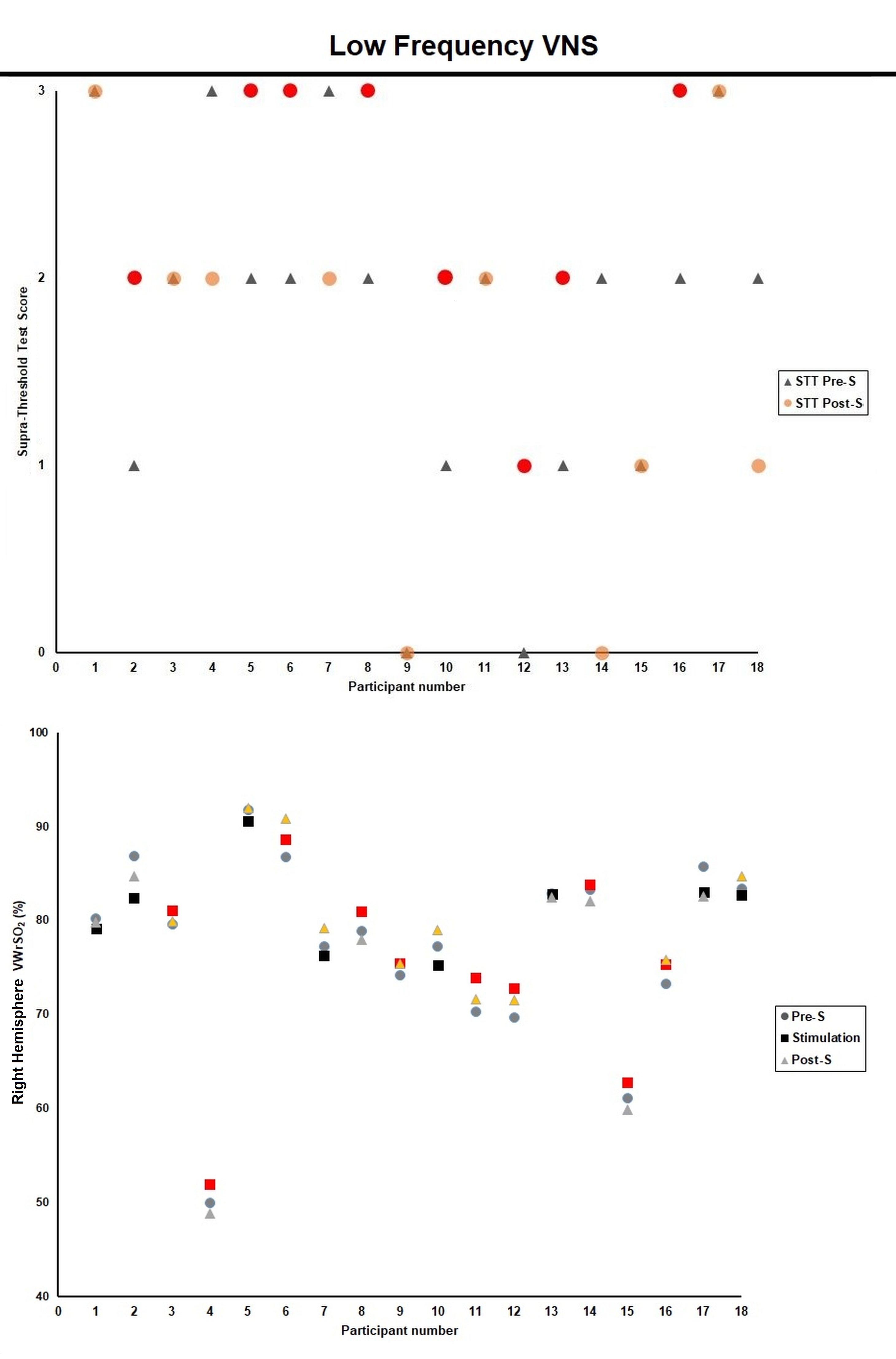

Supplement: Supplementary file 2 [file Image_2.jpeg]

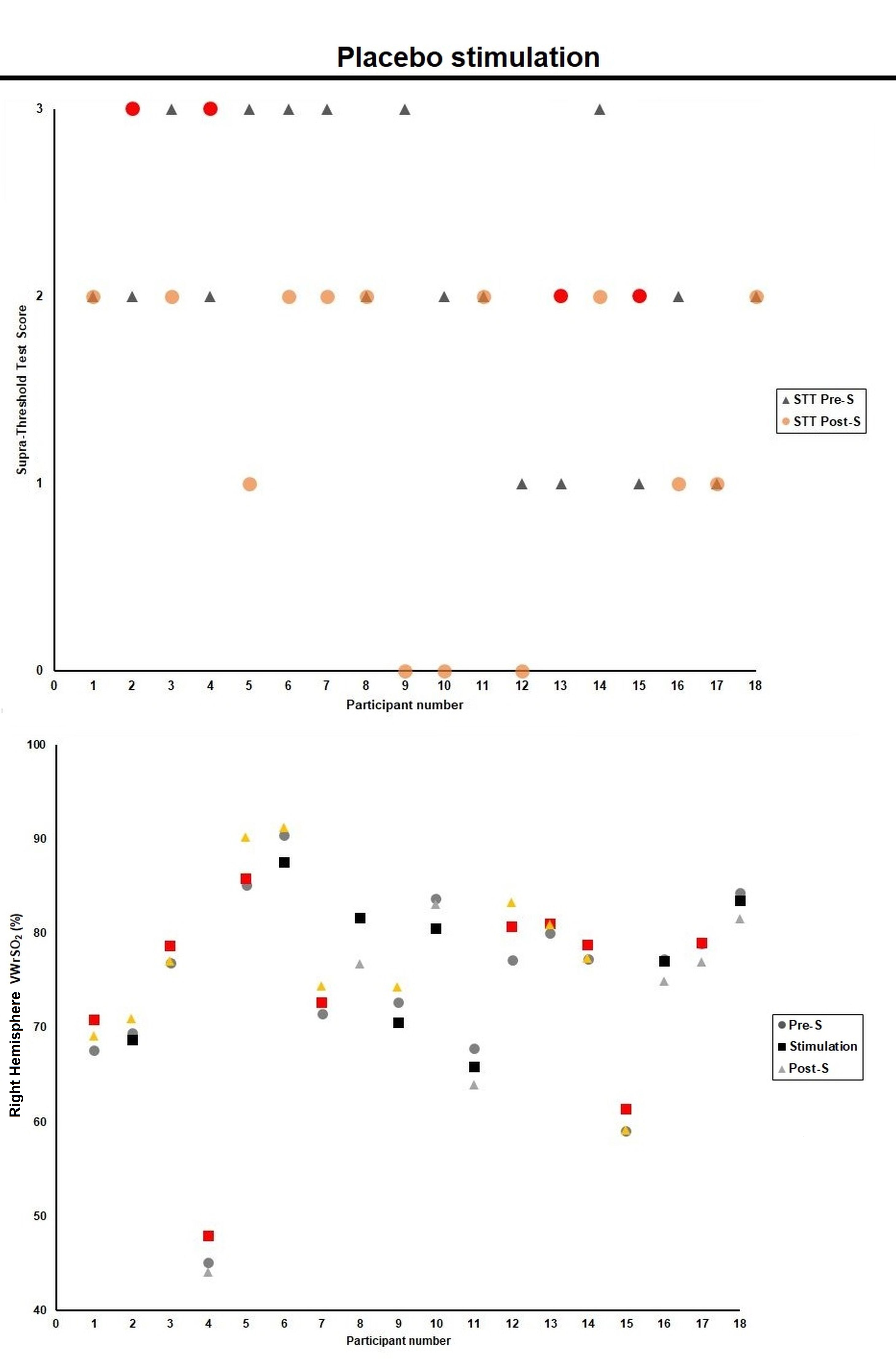

Supplement: Supplementary Files 1–3 — Scatterplot graph which displays each participant's scores, before and after each stimulation parameter (high frequency VNS, low frequency VNS and placebo) for the STT scores (scores range = 0–3) in combination with participant's recordings for all three stages of the experiment (pre-stimulation STT; Pre-S, stimulation and post-stimulation STT; Post-S) for the right hemisphere of the OFC, measuring venous oxygen reserve (VWrSO2 %) using NIRS. In the STT score figures (Figure on top), “Red” color represents participants who improved, while on the STT right hemisphere NIRS recordings (Figure on bottom), “Red” color represents improvements (increased % in VWrSO2) in the stimulation stage from the pre-stimulation stage and “Orange” color represents improvements (increased % in VWrSO2) in the post-stimulation stage from the pre-stimulation stage. Supplementary file 1 shows the results from high frequency VNS, Supplementary file 2 shows the results from the low frequency VNS and Supplementary file 3 shows the results from the placebo stimulation. [file Image_3.jpeg]
